# Association analyses of rare variants identify two genes associated with refractive error

**DOI:** 10.1371/journal.pone.0272379

**Published:** 2022-09-22

**Authors:** Karina Patasova, Annechien E. G. Haarman, Anthony M. Musolf, Omar A. Mahroo, Jugnoo S. Rahi, Mario Falchi, Virginie J. M. Verhoeven, Joan E. Bailey-Wilson, Caroline C. W. Klaver, Priya Duggal, Alison Klein, Jeremy A. Guggenheim, Chris J. Hammond, Pirro G. Hysi, the CREAM Consortium; the UK Biobank Eye; Vision Consortium

**Affiliations:** 1 Department of Ophthalmology, King’s College London, London, United Kingdom; 2 Department of Twins Research and Genetic Epidemiology, King’s College London, London, United Kingdom; 3 Department of Ophthalmology, Erasmus Medical Center, Rotterdam, The Netherlands; 4 Department of Epidemiology, Erasmus MC, Rotterdam, The Netherlands; 5 Computational and Statistical Genomics Branch, National Human Genome Research Institute, National Institutes of Health, Baltimore, Maryland, United States of America; 6 NIHR Biomedical Research Centre at Moorfields Eye Hospital NHS Foundation Trust and the UCL Institute of Ophthalmology, London, United Kingdom; 7 Department of Ophthalmology, St Thomas’ Hospital, Guys and St ’Thomas’ NHS Foundation Trust, London, United Kingdom; 8 Institute of Ophthalmology, University College London, London, United Kingdom; 9 UCL Great Ormond Street Hospital Institute of Child Health, London, United Kingdom; 10 Ulverscroft Vision Research Group, University College London, London, United Kingdom; 11 Department of Ophthalmology, Radboud University Medical Center, Nijmegen, the Netherlands; 12 Department of Epidemiology, Johns Hopkins Bloomberg School of Public Health, Baltimore, MD, United States of America; 13 Department of Oncology, Sidney Kimmel Comprehensive Cancer Center at Johns Hopkins, Johns Hopkins University, Baltimore, Maryland, United States of America; 14 Department of Pathology, Johns Hopkins School of Medicine, Johns Hopkins University, Baltimore, Maryland, United States of America; 15 School of Optometry & Vision Sciences, Cardiff University, Cardiff, United Kingdom; Brigham and Women’s Hospital and Harvard Medical School, UNITED STATES

## Abstract

**Purpose:**

Genetic variants identified through population-based genome-wide studies are generally of high frequency, exerting their action in the central part of the refractive error spectrum. However, the power to identify associations with variants of lower minor allele frequency is greatly reduced, requiring considerable sample sizes. Here we aim to assess the impact of rare variants on genetic variation of refractive errors in a very large general population cohort.

**Methods:**

Genetic association analyses of non-cyclopaedic autorefraction calculated as mean spherical equivalent (SPHE) used whole-exome sequence genotypic information from 50,893 unrelated participants in the UK Biobank of European ancestry. Gene-based analyses tested for association with SPHE using an optimised SNP-set kernel association test (SKAT-O) restricted to rare variants (minor allele frequency < 1%) within protein-coding regions of the genome. All models were adjusted for age, sex and common lead variants within the same locus reported by previous genome-wide association studies. Potentially causal markers driving association at significant loci were elucidated using sensitivity analyses by sequentially dropping the most associated variants from gene-based analyses.

**Results:**

We found strong statistical evidence for association of SPHE with the *SIX6* (p-value = 2.15 x 10^−10^, or Bonferroni-Corrected p = 4.41x10^-06^) and the *CRX* gene (p-value = 6.65 x 10^−08^, or Bonferroni-Corrected p = 0.001). The *SIX6* gene codes for a transcription factor believed to be critical to the eye, retina and optic disc development and morphology, while *CRX* regulates photoreceptor specification and expression of over 700 genes in the retina. These novel associations suggest an important role of genes involved in eye morphogenesis in refractive error.

**Conclusion:**

The results of our study support previous research highlighting the importance of rare variants to the genetic risk of refractive error. We explain some of the origins of the genetic signals seen in GWAS but also report for the first time a completely novel association with the *CRX* gene.

## Introduction

Myopia is a common eye disorder characterised by an imbalance between different refractive components of the eye and axial length [1]. The prevalence rates of myopia and its related complications are on the rise in South East Asia and rapidly increasing in Europe and the US [2]. Both environmental and genetic factors play a role in the pathophysiology of refractive errors (RE). Refractive errors, especially high and pathological myopia, are leading causes of preventable vision loss worldwide and sources of significant ocular complications [1]. Previous studies have shown that individuals with myopia greater than 6 dioptres are at increased risk of other eye conditions and are more susceptible to several sight-threatening complications [1].

Recent genome-wide studies discovered hundreds of distinct loci harbouring genes involved in refraction development [3]. Cumulatively those variants accounted for approximately 18% of total heritability [3], while twin studies estimate the RE heritability between 50%-90% [4]. The missing heritability in refractive error GWAS could be attributed to several causes, such as confounding arising from linkage disequilibrium, statistical power limitations, epistasis and heritability explained by rare genetic variants that are usually not identifiable by traditional genetic association studies. Statistical power to detect associations with individual genetic variants is proportional to the magnitude of the risk they individually confer, but more crucially, to their frequency in the population.

Here we aim to evaluate the impact of lower frequency genetic variation on refractive error in the UK Biobank sub-sample of 50,893 unrelated individuals of European descent.

## Materials and methods

### Study population

The UK Biobank is a cohort of 500,000 volunteers for whom extensive demographic, phenotypic and biomarker data is available. Approximately 23% of individuals (N = 117,279) participated in comprehensive eye examination, including assessment of non-cycloplegic refractive error represented by mean spherical equivalent [5]. The measurements of non-cycloplegic autorefraction were performed by Tomey RC 5000 device (Tomey Corp., Nagoya, Japan) for each eye separately, and the mean spherical equivalent of the two eyes was taken.

The study was conducted with the approval of the North-West Research Ethics Committee (ref. 06/MRE08/65), following the principles of the Declaration of Helsinki. Study participants provided written informed consent.

Full genotypic data was available for all participants who were included in our analyses. Based on a pre-computed Principal Component Analysis, we ascertained individuals’ ancestry identity by state. Only individuals who were of full and homogenenous European ancestry were analysed; in cases where familial relatedness was observed (defined as a PI_HAT > 0.06), only one individual for each pair was included in the analyses.

### Whole exome sequencing data

Approximately 200,629 participants from the UK Biobank cohort were selected for the second tranche of whole-exome sequencing. Individuals who had more complete data, such as baseline measurements, MRI imaging, hospital episodes and primary care records, were prioritised for the sequencing [6]. Although the whole-exome study sample was enriched for clinical outcomes and availability of physical measures, it remained largely representative of the general UK Biobank cohort in terms of demographic characteristics and composition. Exome sequencing was performed as described previously [6]. The panel targeted 39 Mb of the human genome and covered 19,396 genes on autosomal and sex chromosomes, including 4,735,722 variants within the targeted regions, comprising 1,229,303 coding synonymous, 2,498,947 nonsynonymous and 231,631 potential loss of function variants within at least one coding transcript. In addition to targeted regions, some variation in exome adjacent regions was also captured–precisely 9,693,526 nucleotide and indel variants. About 98% of the sequenced coding variants had an allele frequency below 1%. To avert issues related to population structure, we restricted our analyses to a homogeneous sample of European ancestry, as ascertained through a principal component analysis of the directly genotyped variants in the sample, as described before [3].

### The CREAM consortium dataset

CREAM (Consortium for Refractive Error and Myopia) was established in 2011 as a collaboration between studies with data on refractive error which had performed genome-wide association analysis based on SNP arrays. For the current study, we included 10 participating studies with available exome chip data. These studies included: Singapore Indian Eye Study (SINDI), Age-Related Eye Study (AREDS), Rotterdam Study I (RSI), Erasmus Rucphen Family Study (ERF), Estonian Genome Center of the University of Tartu (EGCUT), Finnish Twin Study on Aging (FITSA), Ogliastra, Croatia-Korcula, TwinsUK, Raine eye health study (REHS) and Beaver Dam eye study (BDES).

The phenotypes for all individuals participating in any of the CREAM cohorts were assessed through methodologies similar to those used for the UK Bobank participants [7]. Specifically, refractive error in the CREAM-participating cohorts was measured using autorefraction. All cohorts had been genotyped on either the Illumina HumanExome-12 v 1.0 or v 1.1 array. All cohorts were jointly recalled to obtain a larger sample size of rare variants (here defined as variants with a minor allele frequency (MAF) < 0.01), as recalling genotypes simultaneously across all samples increases the ability to call rare variants with a more discrete distinction between allele calls and sensitivity for low-frequency (high-intensity) loci. All data was recalled using GenomeStudio® v2011.1 (Illumina Inc., San Diego, CA, USA). Nine of these predominantly European CREAM cohorts were combined in a single cohort (N = 11,505), henceforth referred to as the CREAM cohort, for analysis. Because of legal requirements the BDES data (N = 1740) made it impossible to analyse this cohort alongside the rest of cohorts participating in CREAM.

### Statistical analyses

To minimise confounding arising from population structure, the study sample was restricted to 50,893 unrelated UK Biobank participants of European descent. The ancestry and relatedness information was calculated based on the genetic data made available from the UK Biobank [8]. Individuals with European descent were identified by projecting UK Biobank participants onto the coordination of 1000 Genome Project principal components. The genetic data was used to identify related individuals by calculating kinship coefficients for all pairs and third-degree or closer relatives were excluded.

Gene-based analyses were conducted in the optimised SNP-set kernel association test SKAT-O test [9] implemented in the rvtests package [10]. The spherical equivalent measurements were the dependent variable and the weighed allelic burden the independent variables. All analyses were adjusted for age and sex. Our analyses incorporated several variant annotations that previous works have shown to boost the power and accuracy of detecting causal associations in gene-based analyses [11]. Variants in protein-coding regions of genes including synonymous and non-synonymous, stop gain/loss, start gain/loss or splice-site mutations with minor allele frequency below 1% were selected for inclusion. The splicing sites were defined as 3 bases into exon and 8 bases into an intron. Mutations in these regions were annotated as "Normal_Splice_Site" unless they affected the functionally important "GU…AG" region of the intron which was annotated as "Essential_Splice_Site". We excluded UTR variants and polymorphisms with unknown or inconclusive molecular consequences such as intronic variants. We used the GRCh38/hg38 assembly of the human genome (https://www.ncbi.nlm.nih.gov/assembly/GCA_000001405.29) as a reference and variants were identified and annotated using the ANNO package (https://github.com/zhanxw/anno). Gene-based associations with probabilities below the selected Bonferroni multiple testing correction level derived by dividing 0.05 by the total number of 19,293 protein-coding genes that analysed were considered statistically significant (p < 2.59 × 10^−6^). We sought replication of significant genes using the results of the gene-based analysis performed in the predominantly European CREAM cohort, described elsewhere [12] and the BDES cohort described elsewhere [13, 14]. Replication was considered successful if the association probabilities were below the selected Bonferroni multiple testing correction level.

#### Sequential analyses evaluating the role of single variants in gene-based associations

To elucidate which variants were driving observed associations with candidate genes, we performed sequential sensitivity analyses by progressively removing markers from the gene-based analyses. The associations with target genes were assessed using the SKAT-O test adjusted for age, sex and lead common variants within the same locus. The lead common variants were selected from previously published refractive error GWAS [3]. Minor allele frequencies observed were compared with those reported in the gnomAD database [15] and pairwise linkage disequilibrium between any two variants was calculated with reference to the entire European panel included in the “ldlink” online tool (https://ldlink.nci.nih.gov/).

## Results

The study sample included 50893 unrelated UK Biobank participants of European descent; 54% were women with a median age of 57 years (±8 years). Detailed information about the study participants’ demographic and refractive characteristics can be found in Table 1 and characteristics of the spherical equivalent (SPHE) distribution in S1 Fig.

**Table 1:** Characteristics of the study participants.

**Age (mean (SD))**	56.8 (7.9)
**Sex (N, %)**	
Women	27,221 (53.5)
Men	23,672 (46.5)
**SE (mean (SD))**	-0.3 (2.7)
**Refractive status (N, %)**	
Emmetropia	23,193 (32.9)
Hyperopia	13,952 (33.8)
Myopia	13,748 (33.3)

The refractive status was determined based on the measured spherical equivalent and which, for descriptive purposes, we are defining as Emmetropia (spherical equivalent in the range of -1.0 D +1.0 D), myopia (≤-1.0 D) and hyperopia (≥ +1.0D).

**Fig 1:**
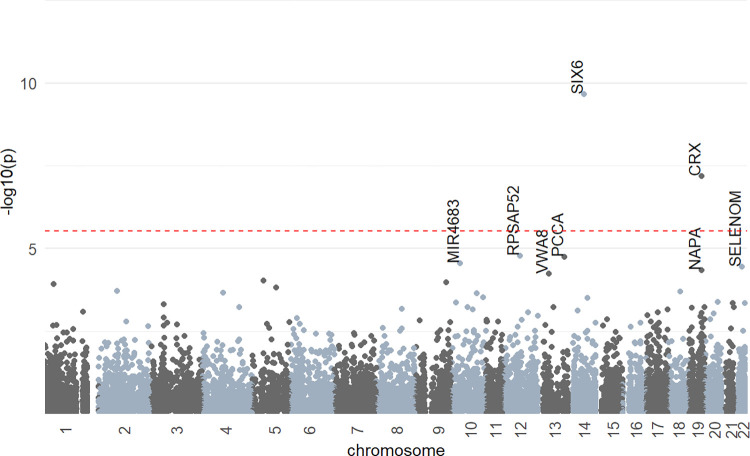
Manhattan plot displaying SKAT-O association results. Each point represents one of the 19,293 genes tested for the association with the spherical equivalent in the UK Biobank cohort (N = 50,893). The plot shows -log10 transformed p-values for each gene plotted against the chromosomal location. The red dashed line indicates the Bonferroni significance threshold (p < 2.59 × 10^−6^). Regions are named with symbols of genes that were most strongly associated with refractive error.

We ran SKAT-O tests using 2,923,839 rare (minor allele frequency, MAF < 0.01) variants in 19,293 genes (Fig 1). The statistically strongest association was observed between SPHE and *SIX6* gene (p-value = 2.15 x10^-10^). The second Bonferroni-significant association was found with *CRX* (p = 6.65 x 10^−08^). This finding was novel and not described in prior refractive error GWAS. Suggestive statistical evidence of association was found for the *RPSAP52* (p = 1.65 x 10^−05^), *PCCA* (p = 1.82 x 10^−05^), *MIR4683* (p = 2.81 x 10^−05^), *SELENOM* (p = 3.52 x 10^−05^), *NAPA* (p = 4.55 x 10^−05^) and *VWA8* (p = 5.68x10^-05^) genes, whose association however did not meet our criteria of statistical significance after multiple testing correction (Table 2).

**Table 2:** Top eight gene associations with refractive error.

Gene symbol	Genetic coordinates	Number of variants	Top SNP at the locus	A1	A1 freq	GnomAD freq	Beta	p-value	Bonferroni corrected p-value
SIX6	14:60509145–60512849	67	14:60509783:G:A	A	0.007	0.0042	-0.86	2.15 x 10^−10^	3.65 x10^-06^
CRX	19:47821936–47843324	92	19:47836338:G:A	A	0.004	0.002	0.71	6.65x 10^−08^	0.001
RPSAP52	12:65758020–65826974	6	12:65825320:G:T	T	3.93x10^-05^		-6.04	1.65x10^-05^	0.28
PCCA	13:100089014–100530437	215	13:100368479:G:T	T	0.0097	0.002	-0.35	1.82x10^-05^	0.31
MIR4683	10:35641171–35641252	4	10:35641193:C:G	G	9.82x10^-06^	4x10^-06^	-11.70	2.81x10^-05^	0.48
SELENOM	22:31104776–31107568	50	22:31105965:T:C	C	1.97x10^-05^		-8.28	3.52x10^-05^	0.6
NAPA	19:47487636–47515063	90	19:47488308:C:T	T	9.82x10^-06^	1.2x10^-05^	-8.99	4.55x10^-05^	0.77
VWA8	13:41566834–41961109	583	13:41891421:C:T	T	9.82x10^-06^	4 x 10^−06^	-14.55	5.68x10^-05^	0.96

Column "Gene symbol" lists the symbols of the genes associated with spherical equivalent. Fields "Genetic coordinates", "p-value" and "Bonferroni corrected p-value" include genetic coordinates (reference genome hg38) of the tested genes, and denote p-values and Bonferroni-corrected p-values for the respective associations. The column "Number of variants" includes the number of tested genetic variants in each respective gene. The field "Top SNP at the locus" lists variants with the statistically strongest associations at the locus. The columns "A1" denotes the effect allele for which betas (Beta) were calculated. The field "A1 freq" shows the frequency of the effect alleles in the study sample and "GnomAD freq" provides the allele frequency from GnomAD database.

No other exome sequencing datasets of comparable size were available to us. However, two smaller cohorts were genotyped for a selection of rare exonic variants using SNP chip arrays, in both of which data from only four of our candidate genes, including *SIX6*, *CRX* and two genes associated with SPHE at suggestive levels in the UK Biobank analysis were available. In addition, only 2 and 3 variants were present in the exome chip data for the *SIX6* and *CRX* genes respectively, that had demonstrated the statistically strongest relationship with spherical equivalent in the discovery cohort. Unsurprisingly given the low minor allele frequencies, none of these variants was in strong linkage disequilibrium with the rare variants that showed significant association with the phenotype in the discovery cohort (R2 < 0.002 for all of them), although they were likely located on the same haplotypes (D’ = 1). None of these genes was associated at statistically significant levels with SHPE in the pooled exome chip cohort from the Consortium for the Refractive Error and Myopia (CREAM, N = 11,505), but we observed a strong association for the *NAPA* gene (SKAT-O p = 3.73 x 10^−05^), in the smaller Beaver Dam Eye Study (BDES, N = 1,740) cohort (Table 3).

**Table 3:** Replication of four loci associated with refractive error using gene-based analyses performed in Beaver Dam (N = 1740) and CREAM Consortium dataset (N = 11,505).

		*Beaver Dam (N = 1740)*		*CREAM (N = 11*,*505)*	
Gene symbol	Genetic coordinates	Number of variants	p-value	Number of variants	p-value
PCCA	13:100089014–100530437	6	0.6	3	0.7
SIX6	14:60509145–60512849	2	0.2	2	0.1
**NAPA**	**19:47487636–47515063**	**1**	**3.73 x 10** ^**−05**^	6	0.2
CRX	19:47821936–47843324	2	0.8	3	0.4

Column "Gene symbol" lists the symbols of the genes associated with spherical equivalent. Fields "Genetic coordinates", "Replication p-value" include genetic coordinates of the tested genes, and denote p-values for the respective associations in Beaver Dam and predominantly European CREAM replication cohorts. The column "Number of variants" includes a number of tested genetic variants in each respective gene. The associations that had p-values below Bonferroni multiple testing correction threshold are shown in bold letters (0.05/5 = 0.01).

To identify independent variants driving gene-based associations at the *SIX6* and *CRX* loci, sensitivity analyses were performed by progressively removing SNPs from gene-based analyses. The removal of rare variants from the gene-based SKAT-O analyses revealed a decrease in the statistical significance of the analyses. The results of these analyses suggested that association with the *SIX6* gene was most strongly influenced by the rs146737847 variant, whose removal resulted in the loss of statistical significance in our samples (S2 Fig). Similarly, exonic marker rs61748438 was identified as a lead variant in the *CRX* locus (S3 Fig). The removal of other functionally important variants within this gene also resulted in a progressive decrease in statistical significance. This gradual decrease may suggest that although gene-based association at both loci is mostly due to the presence of a few lead variants, additional lower frequency variants within these genes may also contribute to associations with spherical equivalent, but our abilities to fully evaluate their role at a general population level may be constrained by sample size and statistical power limitations.

## Discussion

Here we report significant associations between spherical equivalent and rare variants located within *SIX6* and *CRX*, but also *RPSAP52*, *PCCA*, *MIR4683*, *SELENOM*, *NAPA* and *VWA8* genes. In our study, the strongest association was observed with the *SIX6* gene, located on 14q23.1 and which encodes a homeobox protein involved in ocular development [16], morphogenesis and visual perception [17]. The SIX Homeobox 6 (*SIX6*) is part of a group of evolutionarily conserved genes, which were known eye transcription factors [18], which regulate the proliferation of specific retinal cells during optic disc development [18] and retain their importance in the mature retina [18]. *SIX6* is implicated both in the early stages of eye formation and subsequent differentiation of retinal progenitor cells (RPC). Interestingly, previous works have shown that the rs146737847 (Glu129Lys) adversely affects the *SIX6* gene function [19] and is also associated with primary open-angle glaucoma potentially through its known effect over the vertical cup-disc ratio [20]. While observational correlation between glaucoma and myopia status is well known [21, 22] there is little evidence of large-scale shared allelic risk between spherical equivalent and vertical cup-to-disc ratio [3]. The associations observed with both spherical equivalent and glaucoma phenotypes for the rs146737847 suggest that genetic pleiotropy may explain a considerable proportion of the phenotypic correlation between these two common ocular conditions.

The Cone-Rod homeobox gene, or *CRX*, located on 19q13.33, encodes a photoreceptor-specific transcription factor [23]. Although a previous association with refractive error was detected for the broader chromosomal location (1), this is the first time that direct evidence links this gene with spherical equivalent or myopia. This gene is a master regulator of photoreceptor development [24] and differentiation [25]. Certain mutations in this gene cause several retinal disorders, including cone-rod dystrophy, retinitis pigmentosa, adult-onset macular dystrophy and Leber congenital amaurosis [23, 26]. Additionally, the Cone-Rod Homeobox (*CRX*) Transcription Factor regulates the expression of over 700 genes in the retina, including downstream effects over rhodopsin and cone arrestin [27]. CRX expression in the retinal cells was inhibited by light stimulation, a mechanism previously implicated in myopia development [28, 29].

We identified suggestive associations with rare variants located within other genes and SPHE. In particular, our analyses implicate the *NAPA* and *PCCA* genes. Common polymorphisms at genomic loci encompassing these genes are associated with refractive error [3] and the age of refractive correction [7], but this is the first time that, to our knowledge, rare variants within their coding regions are associated with SPHE. *PCAA* encoded the biotin-binding region of mitochondrial Propionyl-CoA carboxylase involved branched and odd chain fatty-acid and cholesterol catabolism [30]. The protein product of the *NAPA* gene is a member of the soluble NSF attachment protein family aiding the fusion and docking of vesicles to target membranes [31]. *NAPA* also participates in synaptic activity and plays a role in neurogenesis [31]. Notably, this particular gene was the only candidate that achieved replication in an independent dataset.

Several novel potential candidates for which we found suggestive evidence of association are implicated in cognitive development and learning difficulty disorders. In particular, Ribosomal Protein SA Pseudogene 52 (*RPSAP52*) is associated with brain structure variations in TWAS [32] and described in genetic investigations of cognitive impairment, neurodevelopmental and neurodegenerative disorders [33]. The polymorphisms within the *RPSAP52* gene were associated with schizophrenia in founder populations [34] and associated with biomarkers of Alzheimer’s disease, such as cerebrospinal fluid beta-amyloid 1–42 levels [35]. Similarly, mutations in microRNA *MIR4683* may be associated with epilepsy in children [36]. Another interesting candidate *VWA8* encodes a mitochondrial ATPase, whose precise function is not fully understood [37]. Genome-wide association studies demonstrated that variation in *VWA8* may influence susceptibility to autism [38] and bipolar disorder [39], and also educational attainment and mathematical ability [40]. *SELENOM*, another novel candidate suggested by our analyses, encoded a selenoprotein that is highly expressed in the brain and that is thought to be essential for normal neurocognitive development [41].

For this study, we used some of the largest ever sample sizes analyzed to date to assess the role of rare variants in refractive error. However, our study has a number of limitations. In our analyses, we assumed a simple dominant model of inheritance, while recessive or compound heterozygosity models of inheritance may also play a role in refractive disorders. Our analyses were restricted to the coding regions of the genome. However, non-coding areas of the genome also proved to be important for several other diseases [42–44] and could potentially provide a new direction for additional myopia work. In our study, we used chip array information in two replication datasets and found relatively little evidence for replication. However, the arrays only include a small number of variants within the exome of the genes of interest and none of which was a particularly strong contributor to the overall gene-based association in the discovery data. This is a general limitation of array-based studies, and to fully validate our results, future work on large scale exome sequencing on independent cohorts will be needed. Additionally, despite the large sample size, statistical power for rare variants is often limited due to the very low allele frequencies. Power will benefit from additional sequencing data from the several national cohorts and biobanks whose data will become available in the future. Finally, the results obtained from an exclusively European population sample are unfortunately not representative of more diverse populations and may not be generalisable to other ancestral groups.

This study demonstrates that variants with significantly large effects on refractive error are extremely rare (Table 2). We identify associations between population spherical equivalent and rare variants located within the protein-coding regions of the *SIX6* gene, which plays an important role in eye morphogenesis and is implicated in several ocular disorders, including myopia. We also identify the *CRX* gene, a transcription factor crucial for the development of photoreceptors, as the origin of an important association signal. Our investigation demonstrates high-quality whole-exome sequencing provides a superior alternative to array-based methods that have power limitations and are prone to bias arising from population admixture [45]. Beyond novel associations, the incorporation of rare variants in existing myopia risk prediction models that currently rely on common polymorphisms will improve their accuracy and augment our understanding of refractive disorders.

## Supporting information

S1 Fig Spherical equivalent distribution in UK Biobank cohort (N = 50,893). The distribution of the spherical equivalent (x-axis) in the samples; the number of participants for each spherical equivalent bin is given in the y-axis.
(PNG)

S2 Fig Sensitivity analyses results for the *SIX6* gene. Y-axis shows the number of *SIX6* variants included in gene-based analyses, testing associations with SPHE. The model was adjusted for age, sex and the best common variant within the same locus. The -log(p-values) from SKAT-O tests are displayed on X-axis.
(PNG)

S3 Fig Sensitivity analyses results for the *CRX* gene. Y-axis shows the number of *CRX* variants included in gene-based analyses, testing associations with SPHE. The model was adjusted for age, sex and the best common variant within the same locus. The -log(p-values) from SKAT-O tests are displayed on X-axis.
(PNG)
