# Supplementary figures and images for: Association analyses of rare variants identify two genes associated with refractive error
Source: PLoS One. 2022 Sep 22;17(9):e0272379. doi: 10.1371/journal.pone.0272379 (PMC9499304; doi:10.1371/journal.pone.0272379)

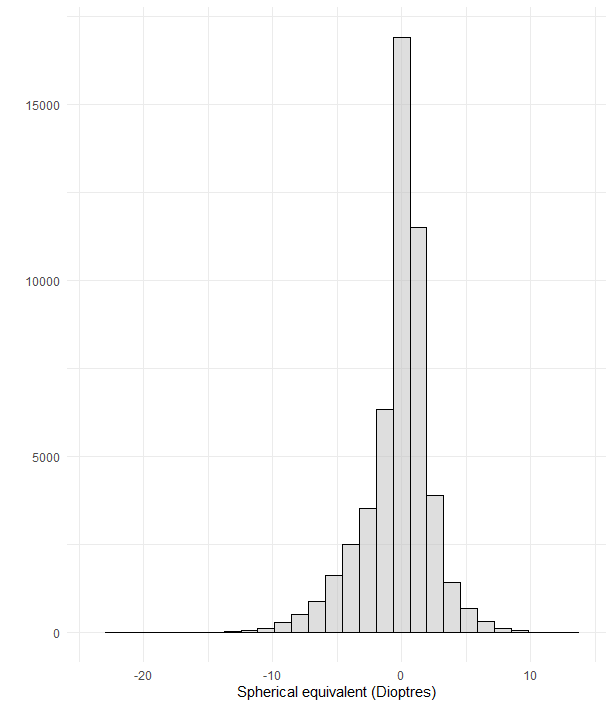

Supplement: S1 Fig — The distribution of the spherical equivalent (x-axis) in the samples; the number of participants for each spherical equivalent bin is given in the y-axis. (PNG) [file pone.0272379.s001.png]

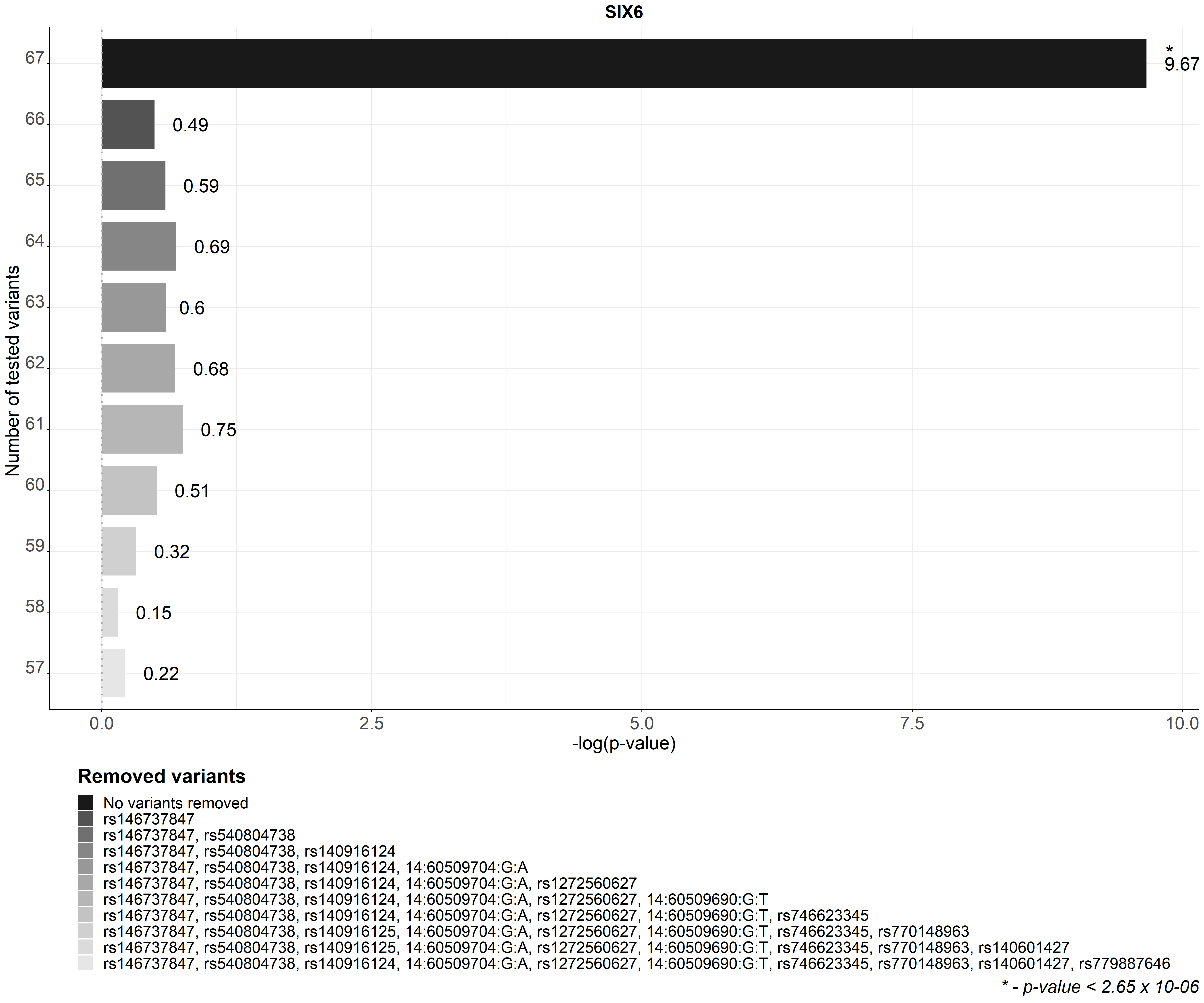

Supplement: S2 Fig — Y-axis shows the number of SIX6 variants included in gene-based analyses, testing associations with SPHE. The model was adjusted for age, sex and the best common variant within the same locus. The -log(p-values) from SKAT-O tests are displayed on X-axis. (PNG) [file pone.0272379.s002.png]

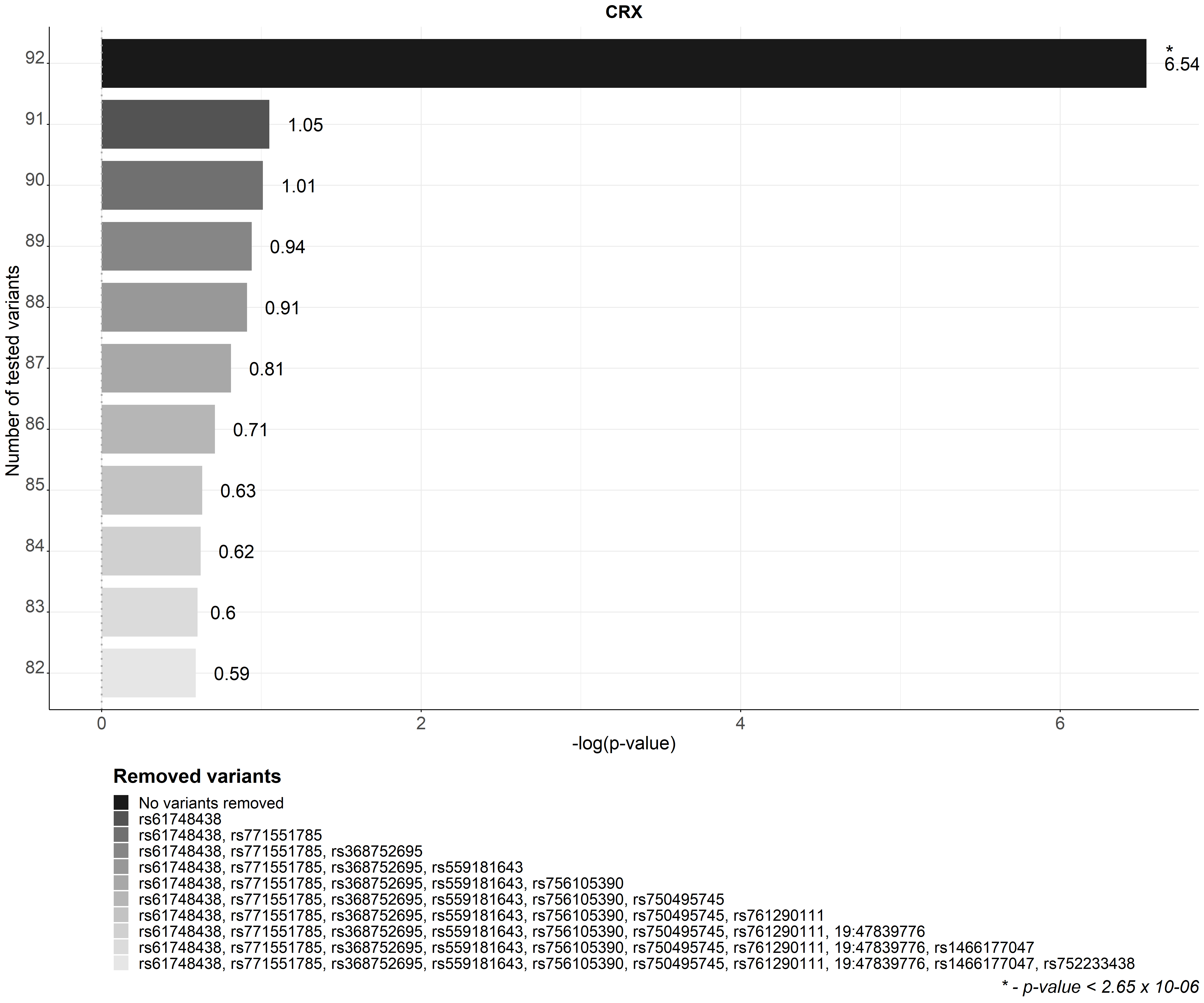

Supplement: S3 Fig — Y-axis shows the number of CRX variants included in gene-based analyses, testing associations with SPHE. The model was adjusted for age, sex and the best common variant within the same locus. The -log(p-values) from SKAT-O tests are displayed on X-axis. (PNG) [file pone.0272379.s003.png]
